# Shaping characteristics of excavation contours in sequential controlled fracture blasting of rock-anchored beams in Shuangjiangkou underground powerhouse

**DOI:** 10.1038/s41598-023-42590-4

**Published:** 2023-09-20

**Authors:** Yanglong Chen, Junhong Huang, Tengsheng Zhang, Zixu Wang, Xinping Li, Yi Luo, Tingting Liu

**Affiliations:** 1 School of Civil Engineering and Architecture, Wuhan University of Technology, Wuhan, 430070 China; 2 School of Resources and Environmental Engineering, Wuhan University of Technology, Wuhan, 430070 China; 3 Sanya Science and Education Innovation Park, Wuhan University of Technology, Sanya, 572024 China

**Keywords:** Engineering, Civil engineering

## Abstract

Influences of high in-situ stress generally need to be considered when excavating deep underground caverns. The dynamic fracture behaviors of rocks under blast loads were investigated by using the rock-anchored beam excavation in underground powerhouses of Shuangjiangkou Hydropower Station in Sichuan Province, China as the engineering background. To solve the problems of the poor blasting breakage effect of rocks and the difficulty in protecting surrounding rocks during excavation, mechanical properties of granite under static and dynamic loads were investigated and the sequential controlled fracture blasting (SCFB) method was adopted during in-situ tests. Based on the Riedel-Hiermaier-Thoma constitutive model and the strength criterion, software LS-DYNA was employed to simulate the dynamic propagation of blasting-induced cracks. The contour shaping effect obtained via numerical simulation is generally consistent with the test results. The results show that SCFB can to some extent control the direction of crack initiation and rock fracture behavior of the blasthole wall cracks and the spacing of successive bursting holes is about 10 times the diameter of the blastholes when the cracks between the blastholes are shaped the best effect. Moreover, the magnitude and direction of principal in-situ stress can both affect the propagation path and length of blasting-induced cracks. The results of the research on the excavation and construction of deeply buried underground caverns have a certain reference value.

## Introduction

With the increasingly more projects to utilize underground spaces, mine underground resources, and develop underground energy in various countries in the world, more stringent technological requirements are set for large-scale construction of underground projects. Due to the complex geological conditions of deep rocks, the rock-breakage mechanism in the blasting excavation process of deep underground engineering has become a technical issue of note in certain professions^1–3^. Deep underground caverns are generally constructed in environments with high in-situ stress and their blasting excavation process is under the superposition of the high static stress field and the blast-induced dynamic stress field. This complicates the propagation and evolution of blasting-induced cracks, making it difficult to control the shaping of the excavation contours^4–6^. Therefore, it is necessary to conduct an in-depth investigation of the precision blasting excavation method for the directional expansion of blasting-induced cracks.

Important structures in deep underground caverns often need to be excavated using fine blasting methods, such as groove-cut borehole, slit charge, binding energy tube, etc. which can guide the expansion of blasting-induced cracks along the direction of the line of the blastholes. The advantages and disadvantages of these excavation methods are shown in Table 1. The SCFB method is not used to increase the scope of other processes, but by changing the time difference between the detonation of charges in adjacent holes to control the expansion of the path of blasting-induced cracks, to a certain extent, can reduce the construction costs, has better potential for application.

**Table 1:** Advantages and disadvantages of traditional precision blasting excavation methods.

Methods	Advantages	Disadvantages
Groove-cut borehole^7^	The cracks induced by groove-cut borehole blasting propagate approximately at a uniform and steady rate between adjacent boreholes. The fluctuation range of the stress intensity factor is relatively small, which facilitates the formation of a smooth contour surface	These methods can guide blast-induced cracks to some extent along the line connecting the boreholes. They require additional construction procedures on the existing borehole layout, thereby increasing economic and time costs
Slit charge^8^	The slit charges play a guiding role in both explosive shock waves and explosive gas. When the non-coupling coefficient is 1.67, it is most conducive to the oriented propagation of blast-induced cracks	
Binding energy tube^9^	Cumulative blasting exerts a directional guiding effect on crack propagation	

Some researchers explored the factors influencing the crack propagation and surrounding rock stability in rock masses. Xue et al.^10,11^ found that after gas intrudes into cracks, the increase in gas pressures can cause rock expansion and reduce the aperture of the cracks. Azarafza et al.^12,13^ investigate rock stability using a crack random generation algorithm and numerical simulation methods. Zhang et al.^14^ revealed the spatio-temporal evolution of a water-conducting fractured zone of overlying strata in the Kongzhuang coal mine. Additionally, other scholars have studied the dynamic crack propagation in rocks under the in-situ stress. For example, Li et al.^15–17^ used the static loading equipment to investigate influences of the in-situ stress on dynamic mechanical behaviors of rocks. Xia et al.^18^ found that the crack propagation velocity is inhibited by the prestress by analyzing the dynamic failure of rock slabs under prestressed conditions. Yang et al.^19^ and Deng et al.^20^ explored influences of the static stress field on the propagation of blasting-induced cracks. They found that the lateral compressive stress orthogonal to the crack propagation direction reduces the stress intensity factor at the crack tip and may hinder crack propagation. Yang et al.^21^ used the finite element software LS-DYNA to reveal the relationship between the in-situ stress and the excavation damaged zone and found that in-situ stress is a main factor for formation of the excavation damaged zone in underground engineering. Yi et al.^22^ studied influences of in-situ stress on rock breakage in the blasting process and concluded that crack propagation near the blastholes is mainly controlled by blast loads while in-situ stress mainly affects the direction of propagation of distant cracks. By conducting tests and simulation, Zhu et al.^23–26^ measured the crack initiation toughness and crack propagation in rocks under in-situ stress and described the fracture behaviors and characteristics of rocks under blast loads. Li et al.^27^ investigated the evolution of seepage in fractured rock masses under mining-induced stress. Xue et al.^28^ studied the crack propagation behavior during the hydraulic displacement fracturing process and found that with the increase of the in-situ stress difference, cracks tend to extend in the direction of the maximum in-situ stress.

These studies show that in-situ stress can, to some extent, influence the crack propagation behaviors while the main factor that changes the dynamic crack propagation is still the blast loads^29–31^. Therefore, some scholars also controlled the propagation paths of blasting-induced cracks based on the delayed exploding time and the effect of uncharged blastholes. For example, Li et al.^32^ proposed an excavation method based on sequential controlled fracture blasting, which reduces damage to surrounding rocks while smoothing the resulting excavation contours. Yue et al.^33^ considered that stress waves generated in the blasthole detonated earlier form a tensile stress field near the wall of blastholes that are blasted later, which is conducive to crack initiation, when the delay time of millisecond blasting meets certain condition. Khandelwal and Singh^34^ discussed the advantage of blasting with precisely-controlled delay time in reducing vibration of surrounding rocks. Shi et al.^35^ studied changes in the decline rate of superimposed waveforms of single blast waveforms with the delay time being of the order of magnitude associated with millisecond blasting. Wang et al.^36^ found strong stress superposition, reflection, and tension of blasting stress waves at uncharged blastholes. Chen et al.^37^ held that uncharged blastholes may affect the tangential stress distribution of cracks and promote crack propagation along the long-axis of uncharged blastholes. Mohanty et al.^38^ found that blasting stress waves are produced by detonation of charged blastholes and then propagate to uncharged blasthole walls, which thus induces a dynamic stress concentration, guiding propagation of blasting-induced cracks. Xie et al.^39^ stated that central blasthole in cut-blasting can serve as a free face. Jayasinghe et al.^40^ used a model combining three-dimensional (3D) coupled fluid dynamics (smoothed particle hydrodynamics (SPH)) and the finite element method (FEM) to show that cracks induced by detonation generally occur along the direction of greatest initial stress. Based on a rock-blasting model, Yang et al.^41^ found that the tangential stress of blasting stress waves propagating to the wall of neighboring blastholes is always greater than the radial stress.

In summary, existing research into the influences of in-situ stress on dynamic propagation behaviors of cracks in rocks and controlling propagation paths of blasting-induced cracks by optimizing the blasting method is sparse, therefore, it is essential to conduct a thorough investigation into rock blasting and excavation under deep and complex geological conditions. The current research took the blasting excavation of rock-anchored beams in underground powerhouses of the Shuangjiangkou Hydropower Station in Sichuan Province, China as a case study. The SCFB method was used to conduct field tests to address the issue of poor contour surface formation in blasting excavation. Additionally, LS-DYNA software was employed to simulate the dynamic propagation behavior of blast-induced cracks under conditions of different borehole spacings and stresses. The results of this study have significant implications for the design and stability analysis of the blasting excavation of underground powerhouses in complex geological environments.

## Engineering background of Shuangjiangkou underground powerhouse

### Engineering overview

**Figure 1:**
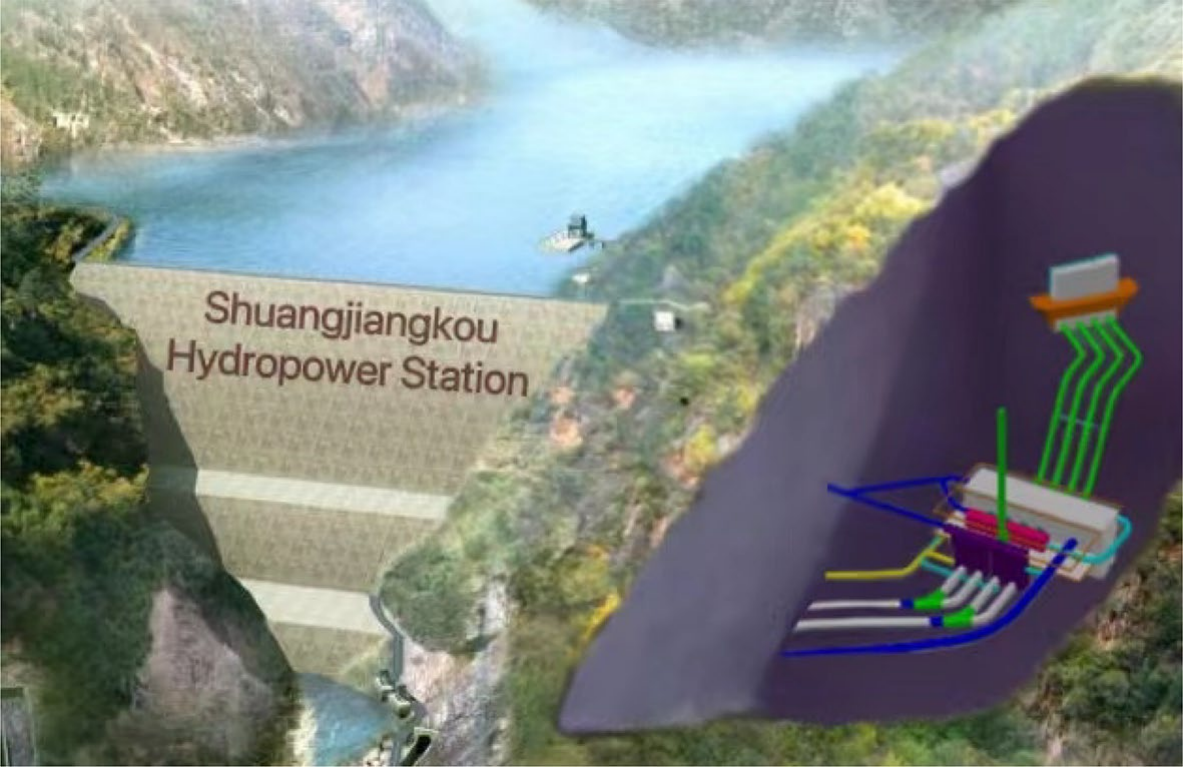
3D perspective of underground caverns of Shuangjiangkou Hydropower Station.

Shuangjiangkou Hydropower Station lies in the Dadu River Basin in Aba Tibetan and Qiang Autonomous Prefecture, Sichuan Province, China, and its control catchment covers an area of 39,330 km^2^, where the average annual runoff is 504 m^3^/s. The underground water diversion and power generation system consisting of the main powerhouse, auxiliary powerhouse, main transformer chamber, and main surge chamber was adopted. The 3D perspective of the underground caverns of Shuangjiangkou Hydropower Station is illustrated in Fig. 1.

**Figure 2:**
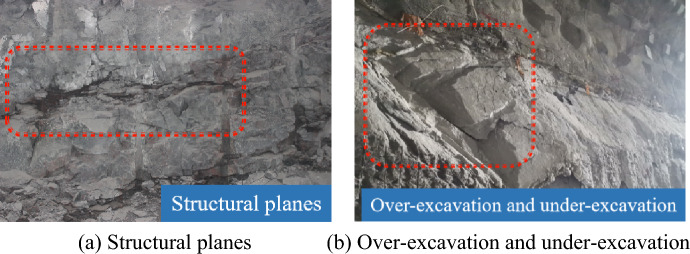
Structural planes in underground rocks and over-excavation and under-excavation at Shuangjiangkou Hydropower Station.

The outcrop at the buildings mainly included fresh porphyritic-like biotite K-feldspar granite. Adit exploration indicated that the rocks were compact, hard, intact, and not cut by regional faults; local dikes were well developed. Some structural planes in rocks and the over-excavation and under-excavation are shown in Fig. 2.

**Figure 3:**
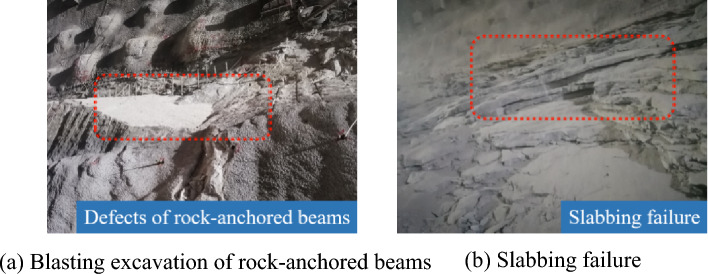
Blasting excavation of rock-anchored beams and slabbing failure.

The locations of rock-anchored beams in underground powerhouses feature high in-situ stress and large blast loads, making it difficult to perform presplitting blasting. In addition, direct blasting is likely to generate many large blocks per unit explosive used, so it fails to reach the expected blasting effect. Considering this, smooth blasting excavation was conducted at rock-anchored beams. Due to development of joint fissures in strata, over-excavation and under-excavation persisted after blasting excavation, and obvious sliding failure occurred in rocks occurring as slabs (Fig. 3).

To solve the problem of the poor blasting excavation effect of rock-anchored beams in underground powerhouses of Shuangjiangkou Hydropower Station, a more elaborate new method to control blasting excavation needs to be developed according to the high in-situ stress and joint distribution characteristics. This is expected to improve the flatness of excavation contours of rock-anchored beams and reduce the damage to the surrounding rock.

### Field tests on mechanical parameters of rock samples

**Figure 4:**
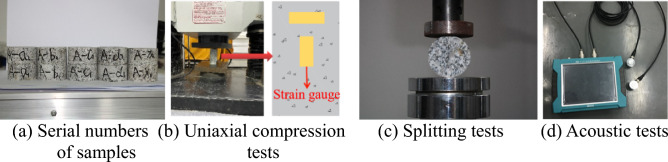
Mechanical testing of granite.

The rocks excavated at underground caverns of Shuangjiangkou Hydropower Station were mainly granite. By in-situ drilling of the local granite, and measuring its mechanical properties, mechanical parameters of rocks needed for establishing the numerical model were obtained (Fig. 4). The tested properties include the density, elastic modulus, compressive strength, Poisson’s ratio, tensile strength, and acoustic velocity.

Rocks collected in the field were prepared into standard cylinders with the diameter *φ* of 50 mm and height *h* of 100 mm and Brazilian disk samples with the diameter *φ* of 50 mm and height *h* of 25 mm. Three rock samples were taken for each test. Each parameter was measured using the following methods: (1) the weighing method was used to measure the density; (2) the compressive strength of rock samples was measured by conducting uniaxial compression tests shown in Fig. 4b; transverse and longitudinal strain gauges were pasted on rock samples to test the transverse and longitudinal deformation of materials, so as to calculate the Poisson’s ratio; (3) the tensile strength of rock samples was tested by conducting the uniaxial Brazilian disk splitting tests shown in Fig. 4c; (4) the acoustic velocity was tested using the test equipment for elastic longitudinal wave velocity.

**Figure 5:**
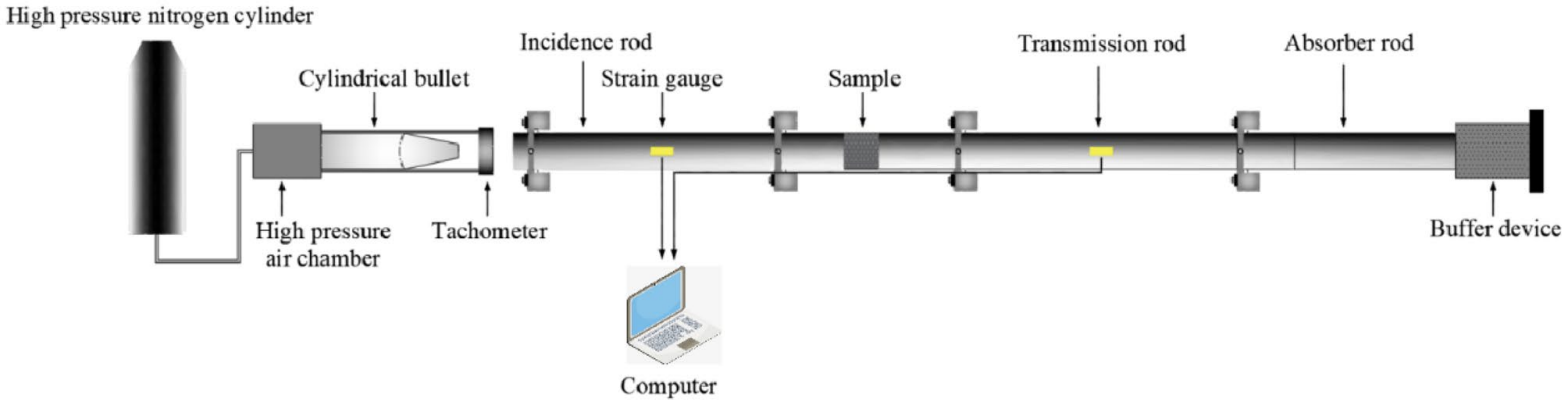
Equipment for split Hopkinson pressure bar tests.

**Figure 6:**
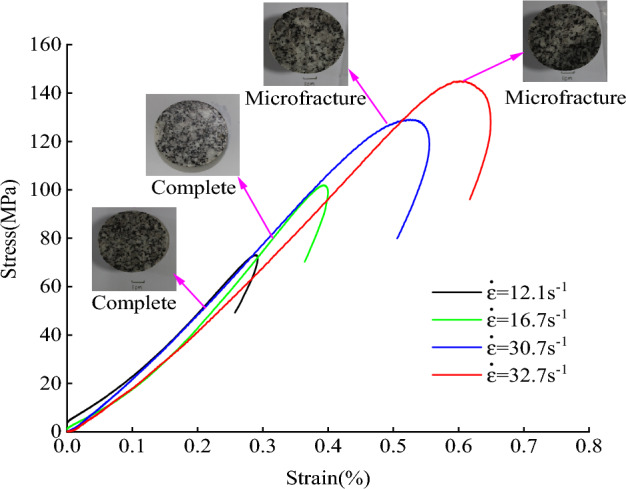
Stress–strain relationship for granite.

Meanwhile, the equipment for the split Hopkinson pressure bar (SHPB) tests in Fig. 5 was adopted to test the stress–strain curves of granite. The stress–strain curves of rock samples under different impact loads are illustrated in Fig. 6. It can be observed that with the increase of the strain rate, microfractures appear in the granite, and the stress–strain hysteresis loop gradually enlarges (it does, however, remain a closed-loop behavior). Mechanical parameters such as the dynamic elastic modulus and the effects of strain rate on granite can be calculated according to the curves.

Mechanical parameters of granite samples separately under static and dynamic loads were obtained by laboratory tests, the experimental results are shown in Table 2.

**Table 2:** Basic mechanical test data (average values).

Specimen	Density (g/cm^3^)	Poisson's ratio	Elastic modulus (GPa)	Compressive strength (MPa)	Tensile strength (MPa)	P-wave velocity (m/s)
Granite	2.73	0.13	144.00	169.85	4.16	6130

## Numerical simulation study

### SCFB

**Figure 7:**
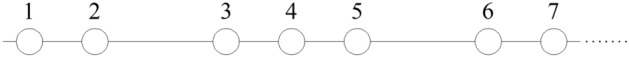
Blastholes arrangement of SCFB.

The SCFB method mainly involves dividing the boreholes into pre-blast holes and post-blast holes in a certain arrangement (Fig. 7). Among them, boreholes 1, 4, and 7 are pre-blast holes, while boreholes 2, 3, 5, and 6 are post-blast holes. The principle is to use the stress waves generated by the pre-blast holes to induce dynamic stress concentration on the borehole walls of the post-blast holes. Subsequently, the post-blast holes are detonated. When the initial cracks are formed along the line connecting the post-blast holes on their borehole walls, the cracks can further propagate under the combined effect of explosive stress waves and blast gases, thus controlling the expansion of cracks in other directions on the borehole walls. This method not only reduces damage to the surrounding rock to some extent but also allows for an increased spacing between adjacent post-blast holes, reduces the number of drill holes, and increases work efficiency.

SCFB proposed by Li et al.^32^ is to enable tensile fracture of rocks around blastholes detonated later along a specific direction under the tensile stress induced by blasting stress waves produced by blastholes detonated earlier on the wall of blastholes detonated later^42–48^. In generally cases, rock failure under uniaxial compression (without or with low confining pressure) is dominated by tensile failure. The failure of micro-elements in materials is caused by tensile deformation, which in turn also causes certain damage. Therefore, the circumferential strain $\varepsilon_{3}$ needs to be used to describe the damage factor *D*^49^.

1 $$ D = \frac{{N_{{\text{F}}} }}{N} = 1 - \exp \left[ { - \left( {\frac{F}{{F_{0} }}} \right)^{m} } \right] = 1 - \exp \left[ { - \left( {\frac{{\varepsilon_{3} }}{{F_{0} }}} \right)^{m} } \right] $$

In this way, the damage constitutive model under tensile failure can be obtained:

2 $$ \sigma_{1} = E\varepsilon_{1} (1 - D) + 2\mu \sigma_{3} = E\varepsilon_{1} \exp \left[ { - \left( {\frac{{\varepsilon_{3} }}{{F_{0} }}} \right)^{m} } \right] + 2\mu \sigma_{3} $$

When considering the plain strain, Yi^50^ took transient waves excited by uniform pressure $p(t)$ on internal walls of the cylindrical cavity with a radius of $a$ in an infinite, uniform, elastic medium as an example. Cylindrical coordinates $(r,\theta ,z)$ were used to deduce the relationship between stress and potential at detonation of single cylindrical blastholes.

3 $$ \left\{ \begin{gathered} \begin{array}{*{20}l} {\sigma_{r} (r,t) = \lambda \nabla^{2} \varphi + 2\mu \frac{{\partial^{2} \varphi }}{{\partial r^{2} }}} \hfill \\ {\sigma_{\theta } (r,t) = \lambda \nabla^{2} \varphi + (2\mu /r)\frac{\partial \varphi }{{\partial r}}} \hfill \\ {\sigma_{z} (r,t) = v\left( {\sigma_{r} + \sigma_{\theta } } \right)} \hfill \\ \end{array} \hfill \\ \frac{{\partial^{2} \varphi (r,t)}}{{\partial r^{2} }} + \frac{1}{r}\frac{\partial \varphi (r,t)}{{\partial r}} = \frac{{\partial^{2} \varphi (r,t)}}{{c_{p}^{2} \partial t^{2} }}\quad (r > a,t > 0) \hfill \\ \end{gathered} \right. $$

For convenience in such unified research into the stress state of different blasthole walls, the coordinates can be converted into the Cartesian coordinate system. Assuming the *X* and *Y*-axes in the Cartesian planar coordinate system are separately along and vertical to the direction of the connecting line of blastholes, then Eq. (3) can be rewritten as

4 $$ \left\{ {\begin{array}{*{20}l} {\sigma_{{\text{s}}} = \sigma_{r} \cos^{2} \phi + \sigma_{\theta } \sin^{2} \phi } \hfill \\ {\sigma_{b} = \sigma_{r} \sin^{2} \phi + \sigma_{\theta } \cos^{2} \phi } \hfill \\ \end{array} } \right. $$

where $\phi$ represents the azimuth in the polar coordinate system; $\sigma_{{\text{s}}}$ and $\sigma_{b}$ denote the radial stress and tangential stress, respectively.

**Figure 8:**
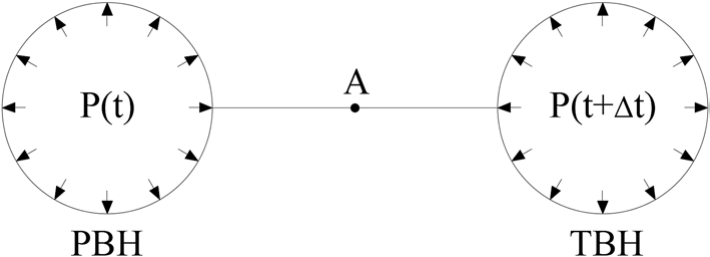
Stress action in blastholes detonated successively.

As shown in Fig. 8, when adjacent blastholes are detonated simultaneously, the wave fronts formed by the two blastholes encounter at point A on the connecting line of blastholes, and then the superposition effect of stress waves appears. If delayed blasting is performed for adjacent blastholes, the encountering point and superposition of blasting wave fronts after detonation of adjacent blastholes can be obtained based on the propagation velocities of longitudinal and transverse waves in rocks.

**Figure 9:**
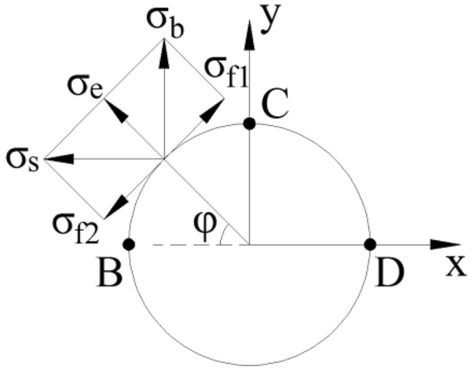
Stress on the wall of uncharged blastholes.

Before detonation of the blasthole detonated later, the blasthole is equivalent to an uncharged blasthole with regard to blasting stress waves produced by the blasthole detonated earlier. Stress distribution on elements of the wall of the blasthole detonated later under the blasting stress waves $\sigma_{s}$ and $\sigma_{b}$ produced by the blasthole detonated earlier is illustrated in Fig. 9, therefore, the radial $\sigma_{e}$ and tangential $\sigma_{f}$ on elements of the uncharged blasthole wall are given by

5 $$ \left\{ {\begin{array}{*{20}c} {\sigma_{e} = \sigma_{b} \sin \varphi + \sigma_{s} \cos \varphi } \\ {\sigma_{f} = \sigma_{b} \cos \varphi + \sigma_{s} \sin \varphi } \\ \end{array} } \right. $$

According to the design principle of SCFB, when the tangential tensile stress $\sigma_{f}$ on element B of the blasthole wall is maximum, that is, $\varphi = 0$ °C and $\sigma_{f} { = }\sigma_{b}$, initial cracks are most likely to develop along the direction of connecting lines of blastholes after detonation of the blasthole detonated later.

### Numerical simulation

Based on the experimental data obtained and referring to previous research findings^51^, the mechanical parameters of the Shuangjiangkou granite were determined (Table 3). Numerical simulation studies on tunnel contour excavation shaping were then conducted to determine the dynamic crack propagation behavior under the action of SCFB.

**Table 3:** Mechanical parameters of granite at Shuangjiangkou Hydropower Station.

Parameter	Parameter meaning	Value	Parameter	Parameter meaning	Value
ρ_0_	Density of material	2730kg/m^3^	A	Failure surface parameter	2.618
α_0_	Initial porosity	1.0	n	Failure surface index	0.7985
P_el_	Initial crushing pressure	113.2MPa	Q_0_	Tension-pressure meridional ratio parameter	0.567
P_comp_	Complete crushing pressure	5.1GPa	B	Rhodes Angle correlation coefficient	0.0105
N	Index of porosity	3.0	β_c_	Shrinkage strain rate index	0.032
A_1_	Ugonian coefficient	40GPa	β_t_	Tensile strain rate index	0.036
A_2_	Ugonian coefficient	0	ε_0_^c^	Refer to compression strain rate	3e8
A_3_	Ugonian coefficient	0	ε_0_^t^	Refer to tensile strain rate	3e9
B_1_	Equation of state parameter	1.22	ε^c^	Failure compression strain rate	3e22
B_2_	Equation of state parameter	1.22	ε^t^	Failure tensile strain rate	3e22
B_3_	Equation of state parameter	1.22	g_c_*	Compression yield surface parameters	1.0
T_1_	Equation of state parameter	40GPa	g_t_*	Tensile yield surface parameters	0.7
T_2_	Equation of state parameter	0	ξ	Shear modulus reduction coefficient	0.5
ƒ_c_	Uniaxial compressive strength	169.85MPa	A_ƒ_	Residual stress intensity parameter	0.873
ƒ_t_*	Tensile/compressive strength ratio	0.0245	n_ƒ_	Residual stress intensity index	0.559
ƒ_s_*	Shear/compressive strength ratio	0.234	D_1_	Initial damage parameter	0.04
G	shear modulus	27.77GPa	D_2_	Parameter of damage	1.0
ε_p_^m^	Minimum failure strain	0.001			

**Figure 10:**
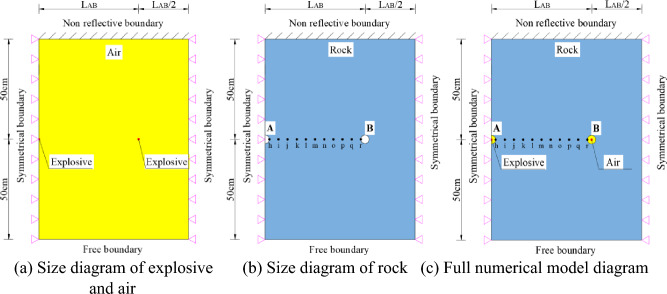
Simplified three-dimensional computational model.

To ensure that the explosive energy in the blastholes is fully transmitted to the rock mass, the air medium elements are completely overlaid with the rock-medium elements in the numerical model, and their sizes are consistent with those of the rock-medium elements. The mesh sizes of different material elements in the numerical model are close to those of the surrounding elements near the blastholes to realize better simulation of the damage between adjacent blastholes. Consequently, the established numerical model contains a relatively large number of elements, typically ranging from 50,000 to 330,000 elements per model. In practical engineering blasting excavation operations, there may be more than a dozen (or even dozens) of blastholes. If all the blastholes are explicitly modelled, the number of elements would be in the millions, making it difficult to perform fluid–structure coupling calculations. Therefore, the model is established under symmetrical boundary conditions on the left and right sides, non-reflective boundary conditions on the upper side, and free boundary conditions on the lower side. The simplified three-dimensional computational model is shown in Fig. 10.

The material model selected for the explosive adopts the *MAT_HIGH_EXPLOSIVE_BURN constitutive model provided in the LS-DYNA program. The explosion products were described using the Jones-Wilkins-Lee (JWL) state equation, which has the general form of:

6 $$ P = A\left( {1 - \frac{\omega }{{R_{1} V}}} \right)e^{{ - R_{1} V}} + B\left( {1 - \frac{\omega }{{R_{2} V}}} \right)e^{{ - R_{2} V}} + \frac{{\omega E_{0} }}{V} $$

where, $P$ represents the detonation pressure, $V$ denotes the relative volume of detonation products, $E_{0}$ signifies the specific internal energy of the explosive per unit volume, and $A$, $B$, $R_{1}$, $R_{1}$ and $\omega$ are constants.

The values of the explosive parameters and the JWL state equation in the blasting model are listed in Table 4.

**Table 4:** Parameters of the explosive and JWL state Equation ^32^.

$\rho$/(g/cm^3^)	$D$/(m/s)	$A$/(Gpa)	$B$/(Gpa)	$R_{1}$	$R_{2}$	$\omega$	$E_{0}$/(J/m^3^)
1.0	3200	214	0.18	4.15	0.95	0.13	4.0 × 10^9^

Herein, $\rho$ represents the density of the explosive, and $D$ denotes the detonation wave velocity.

The air elements are modelled using the *MAT_NULL model from the material model library in LS-DYNA. The equation of state for the *MAT_NULL model is described using the Gruneisen equation.

7 $$ P = \frac{{\rho_{2} C^{2} \mu \left[ {1 + \left( {1 - \frac{{\gamma_{0} }}{2}} \right)\mu - \frac{{\mu^{2} }}{2}} \right]}}{{\left[ {1 - \left( {S_{1} - 1} \right)\mu - S_{2} \frac{{\mu^{2} }}{\mu + 1} - S_{3} \frac{{\mu^{3} }}{{(\mu + 1)^{2} }}} \right]^{2} }} + \left( {\gamma_{0} + \alpha \mu } \right)E_{2} $$

In the equation, $\rho_{2}$ represents the density of the air; $\gamma_{0}$ is the Gruneisen parameter; $\alpha$ is the first-order volume correction of $\gamma_{0}$; $C$ represents the curve intercept; $S_{1}$, $S_{2}$, and $S_{3}$ are coefficients of the curve slope; and $\mu$ is the volume correction. The values of the air material parameters are listed in Table 5.

**Table 5:** Parameters of the air material ^52^.

$\rho_{2}$/(g/cm^3^)	$C$	$S_{1}$	$S_{2}$	$S_{3}$	$\gamma_{0}$	$E_{2}$/(J/m^3^)
0.00125	0.344	0	0	0	1.4	0

The RHT constitutive model was used to simulate the smooth blasting of the tunnel contour. As displayed in Fig. 10, the blasthole diameter *d* is 40 mm, the linear charge density in blastholes is 150 g/m, the equivalent cartridge diameter is 10 mm, and those mechanical parameters of granite at Shuangjiangkou Hydropower Station in Table 3 are used for the rocks. The blastholes detonated earlier and later are separately labelled A and B, and the spacing between adjacent blastholes is 0.5 *d* ≤ *L*_AB_ ≤ 3 *d*. That is, the values of *L*_AB_ are 200, 300, 400, 500, 600, 700, 800, 900, 1000, 1100, and 1200 mm. Considering the precision of commercially available electronic detonators and for the convenience of applying test results to practical engineering, the delay time of detonation of blastholes detonated successively is set to 1 ms and the simulation runs for 2 ms.The units of the numerical model are cm/g/μs.

**Figure 11:**
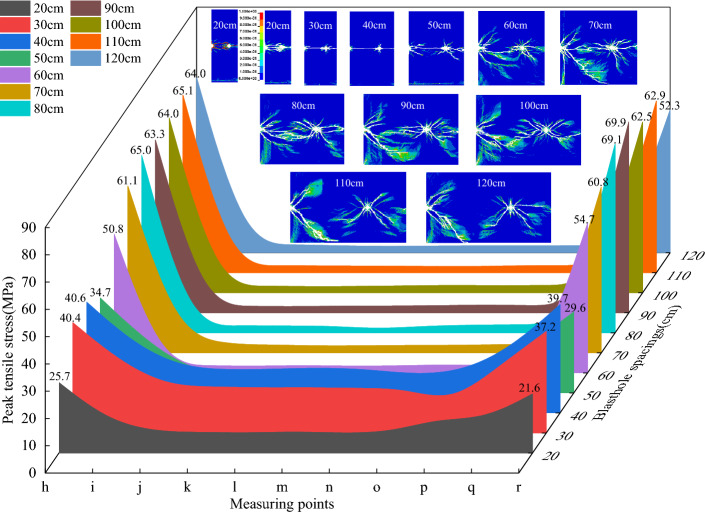
Numerical simulation results of SCFB.

To investigate the propagation of blasting-induced cracks by combining with the stress state of rock elements, 11 measuring points labelled successively from h to r from left to right of the model were uniformly selected on the connecting line of blastholes. In this way, the peak tensile stress at each measuring point on the connecting line of blastholes with different spacings and the numerical simulation results of SCFB were obtained, as shown in Fig. 11.

For the convenience of observing the crack propagation, taking the blasthole spacing *L*_AB_ of 200 mm as an example, it is defined herein that rocks with a damage value exceeding 0.7 were deemed to have failed. Figure 11 indicates that, if *L*_AB_ is not greater than 500 mm, the peak tensile stress at measuring points in the middle is always greater than the tensile strength (4.16 MPa) of granite. Under such conditions, damage to the rock samples mainly occurs along the direction of the connecting line of blastholes. The peak tensile stress in the middle is the maximum and the contour shaping effect is best when *L*_AB_ is 400 mm. This indicates that the case when the spacing of blastholes detonated successively is 10*d* is most favorable to the formation of straight and coalesced fractures.

As *L*_AB_ is increased from 600 to 800 mm, the peak tensile stress at measuring points in the middle is in the range of 2–4 MPa, which is smaller than the tensile strength of granite, and coalesced fractures still can be formed between blastholes; however, not all such fractures are along the direction of the connecting line of blastholes. Over-excavation and under-excavation phenomena still occur and the damage to surrounding rocks is gradually intensified. If the blasthole spacing *L*_AB_ is greater than, or equal to, 900 mm, it is generally difficult to form coalesced fractures along the direction of the connecting line of blastholes and the damage to surrounding rocks is further intensified. This finding indicates that when remaining other blasting parameters unchanged, if the spacing between adjacent blastholes is too large, the superposition effect between different blasting stress waves is weakened and greatly affected by transmission and reflection of stress waves. Consequently, coalesced fractures along the direction of the connecting line of blastholes cannot be formed.

**Figure 12:**
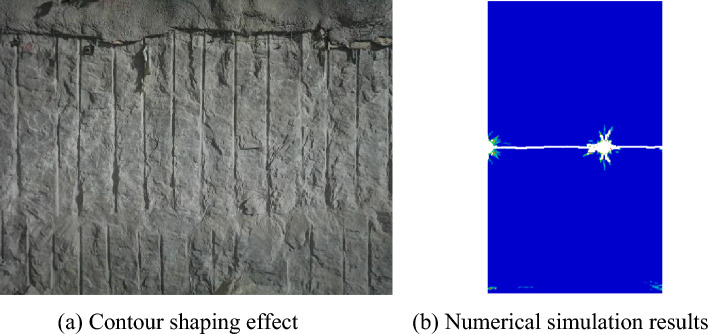
Post-blasting effect with a borehole spacing of 10 times the borehole diameter.

Based on the actual engineering conditions of the underground cavern at the Shuangjiangkou Hydropower Station, the borehole spacing was set to be 10 times the borehole diameter in the blasting excavation test section. As illustrated in Fig. 12, the boreholes can form straight, coalesced cracks, resulting in a satisfactory contour shaping effect. This observation is in good agreement with the numerical simulation results, confirming the accuracy of the numerical simulation.

## Influences of in-situ stress on contour shaping in smooth blasting

Theoretical derivation and numerical simulation results in Section “Numerical simulation study” both imply that detonation timing sequence of blastholes detonated successively can, to some extent, control the crack initiation direction and propagation between adjacent blastholes; because deep underground caverns are generally constructed in high in-situ stress fields, the omnipresent in-situ stress may affect the propagation of blasting stress waves and guide the crack propagation. Therefore, the section further discusses the influences of in-situ stress on the contour shaping effect in smooth blasting based on the RHT constitutive model.

### Secondary stress state of the tunnel under in-situ stress

**Figure 13:**
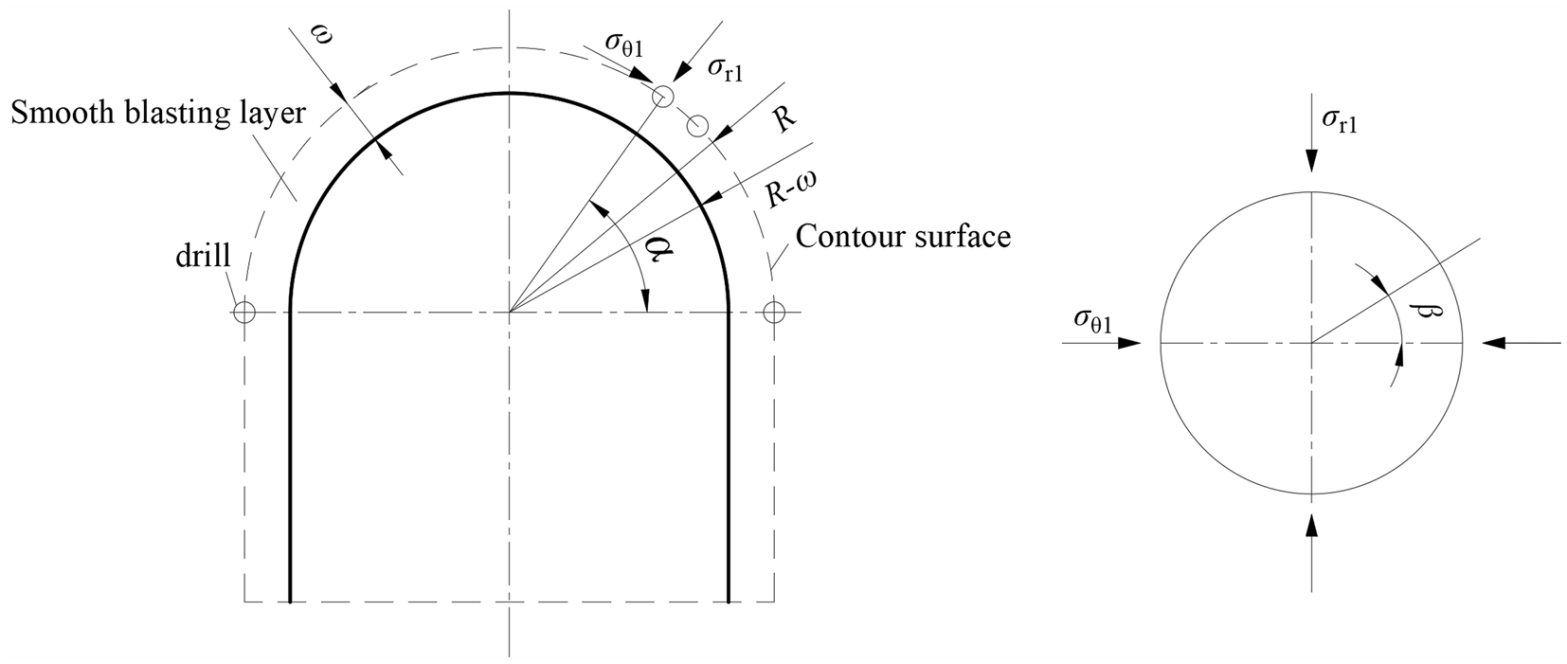
Static in-situ stress on peripheral blastholes under smooth blasting.

Under smooth blasting conditions of the deep tunnel, peripheral blastholes are detonated after blasting of other blastholes on the tunnelling face. The static stress distribution on rocks around peripheral blastholes is displayed in Fig. 13. The formation of coalesced fractures between blastholes arises from joint action of the static secondary stress caused by in-situ stress and the super-dynamic stress induced by detonation of blastholes.

If blastholes on the tunnelling face are detonated while peripheral charged blastholes are not detonated, radial stress $\sigma_{r1}$ and tangential stress $\sigma_{\theta 1}$ with the center of the tunnel (the upper part of which is a semicircle) as the origin of polar coordinates occur at the blastholes due to stress redistribution in surrounding rocks of the tunnel caused by blasting excavation. According to the elastic mechanics of stress redistribution around circular caverns, the shear stress $\tau_{r\theta 1}$ induced by $\sigma_{r1}$, $\sigma_{\theta 1}$, and in-situ stress is

8 $$ \left\{ \begin{gathered} \sigma_{r1} = \frac{{P_{0} }}{2}\left[ {(1 + k)(1 - \frac{{\left( {R_{0} - \omega } \right)^{2} }}{{R_{0}^{2} }}) - (1 - k)(1 - \frac{{4\left( {R_{0} - \omega } \right)^{2} }}{{R_{0}^{2} }} + \frac{{3\left( {R_{0} - \omega } \right)^{4} }}{{R_{0}^{4} }})\cos 2\alpha } \right] \hfill \\ \sigma_{\theta 1} = \frac{{P_{0} }}{2}\left[ {(1 + k)(1 + \frac{{\left( {R_{0} - \omega } \right)^{2} }}{{R_{0}^{2} }}) + (1 - k)(1 + \frac{{3\left( {R_{0} - \omega } \right)^{4} }}{{R_{0}^{4} }})\cos 2\alpha } \right] \hfill \\ \tau_{r\theta 1} = - \frac{{P_{0} }}{2}(1 - k)(1 + 2\frac{{\left( {R_{0} - \omega } \right)^{2} }}{{R_{0}^{2} }} - 3\frac{{\left( {R_{0} - \omega } \right)^{4} }}{{R_{0}^{4} }})\sin 2\alpha \hfill \\ \end{gathered} \right. $$

where $P_{0}$ is the vertical component of in-situ stress; $k$ denotes the lateral pressure coefficient; $R_{0}$ denotes the tunnel radius; $\omega$ represents the thickness of the smooth blasting layer; $\alpha$ is the included angle between the blasthole and the horizontal line.

If the lateral pressure coefficient is $k = 1$, Eq. (8) is rewritten as

9 $$ \left\{ \begin{gathered} \sigma_{r1} = P_{0} \left[ {1 - \frac{{\left( {R_{0} - \omega } \right)^{2} }}{{R_{0}^{2} }}} \right] \hfill \\ \sigma_{\theta 1} = P_{0} \left[ {1 + \frac{{\left( {R_{0} - \omega } \right)^{2} }}{{R_{0}^{2} }}} \right] \hfill \\ \end{gathered} \right. $$

Equations (8) and (9) do not consider influences of perimeter blastholes on the stress field in surrounding rocks (namely, the secondary stress). If there are perimeter blastholes, they inevitably induce stress redistribution in rocks. When $k = 1$, the radial stress $\sigma_{r2}$ and shear stress $\tau_{r\theta 2}$ produced by the stress redistributed again (tertiary stress) in the vicinity of blastholes are 0, while the tangential stress is^53^:

10 $$ \sigma_{\theta 2} = \left( {\sigma_{r1} + \sigma_{\theta 1} } \right) + 2\left( {\sigma_{r1} - \sigma_{\theta 1} } \right)\cos \left( {2\beta } \right) $$

where $\beta$ denotes the included angle between the line connecting the center of the blasthole and any point on the blasthole wall and the perimeter blastholes (Fig. 10).

The excavated radius of the tunnel is much greater than the thickness of the smooth blasting layer, so $\sigma_{r1} < \sigma_{\theta 1}$ at perimeter blastholes, so tensile stress may be generated on blasthole walls in the direction of the line connecting each blasthole ($\beta = 0^\circ ,\,180^\circ$). The value of tensile stress is directly proportional to the in-situ stress. In the case of a large in-situ stress, the tensile stress will exceed the tensile strength of rocks and therefore induce pre-cracks along the direction of the connecting line of blastholes, which conducive to obtaining a good contour-shaping effect.

**Figure 14:**
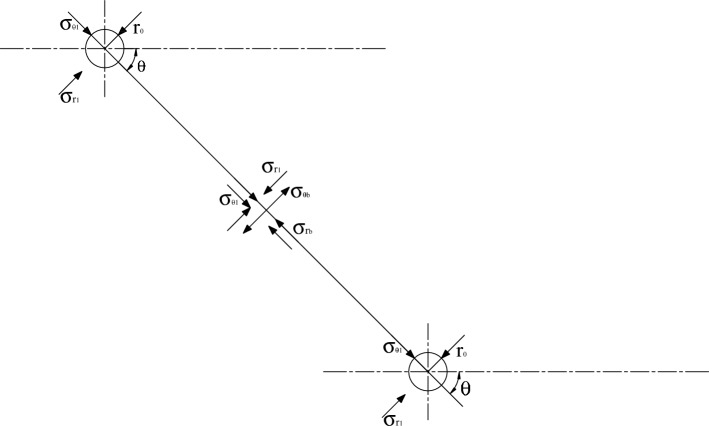
Stress distribution between blastholes under smooth blasting.

At positions far from blastholes on the connecting line of blastholes, the static principal stress in rocks is the redistributed stress (secondary stress) induced by excavation of the tunnel. The direction of the principal stress is always along or vertical to the direction of the connecting line of blastholes. This is consistent with the direction of the blast-induced dynamic stress field (Fig. 14, in which $\sigma_{rb}$ and $\sigma_{\theta b}$ are dynamic stresses caused by blasting). Therefore, the presence of in-situ stress during smooth contour blasting of tunnels is conducive to formation of coalesced fractures along the direction of the connecting line of blastholes and obtaining a smooth blast contour.

### The contour shaping effect under different in-situ stress conditions

Six conditions with the smooth blasting layer of 0.5 m thick and initial in-situ stresses of 0, 4, 8, 16, 24, and 32 MPa were taken as examples to estimate influences of different initial in-situ stress conditions on crack propagation under SCFB. A planar blasting analysis model was established, in which the blasthole diameter *d* is 40 mm. To embody the guiding effect of SCFB on propagation paths of blasting-induced cracks, the adjacent blasthole spacing *L* was set to 800 mm combined with the results in Section “Numerical simulation study” to evaluate the contour-shaping effect under smooth blasting excavation.

**Figure 15:**
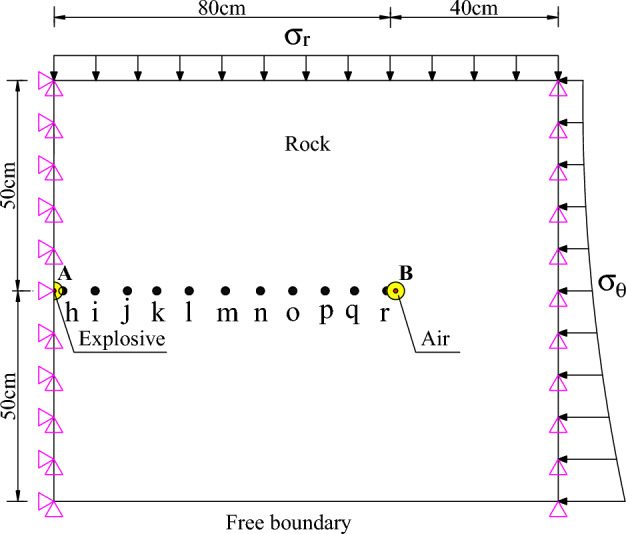
Calculation model for SCFB under different in-situ stress conditions.

The circumferential stress is applied to the right boundary while the radial stress is applied to the upper boundary of the model. Constraints in the *x* and *y*-directions are applied simultaneously to the left boundary, while only constraints in the y direction are applied to the right boundary of the model. Under the condition, the two-dimensional (2D) figure of the calculation model is shown in Fig. 15. Similarly, eleven measuring points were set uniformly on the connecting line of two blastholes and labelled h to r from left to right of the model, to obtain the peak tensile stress at each measuring point under different in-situ stress conditions.

**Figure 16:**
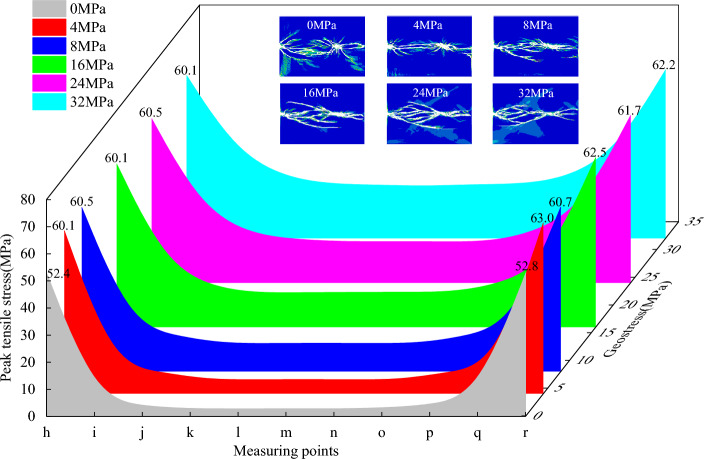
Peak tensile stress at measuring points on connecting line of blastholes under different in-situ stress conditions.

Figure 16 illustrates that the peak tensile stress on elements between blastholes follows the same trend under different initial in-situ stress conditions. Under the same initial in-situ stress conditions, the peak tensile stress decreases with the increasing distance of measuring points from the blastholes. This is because the energy of blasting stress waves gradually attenuates with distance in the transmission process.

The peak tensile stress on elements between blastholes is always small and the crack propagation paths between blastholes are also dispersed when the in-situ stress is 0 MPa. As a result, the shaping effect of excavation contours is poor. If the in-situ stress is 4 and 8 MPa, straight, coalesced fractures can be formed between blastholes, which suggests that SCFB can further enhance the guiding effect on crack propagation under low in-situ stress. As the in-situ stress is further increased to 16, 24, and 32 MPa, the confinement and compression of in-situ stress on rocks are also enhanced. Therefore, blasting-induced cracks propagate along multiple stress concentration paths under joint action of the extrusion of large in-situ stress and the reflection and tension of free faces. In addition, the propagation paths of blasting-induced cracks are similar, which forms wide, criss-cross fractures, leading to over-excavation and under-excavation between blastholes, which directly affects the crack propagation and coalescence effect between blastholes. This finding indicates that when the in-situ stress exceeds a certain value, its influences on the contour shaping effect of rock-anchored beams in underground powerhouses remain unchanged, making it necessary to change parameters including the blasthole spacing, delayed exploding time, and charge structure to improve the smoothness of the resulting excavation contours.

## Conclusion

Based on SCFB, the LS-DYNA software was used to simulate the blasting excavation of underground caverns in the Shuangjiangkou Hydropower Station. The shaping effects of excavation contours of rock-anchored beams under different borehole spacing and stress conditions were compared. The main conclusions are drawn as follows:

1. The spacing between the pre-blast and post-blast holes can influence the initiation direction and propagation path of cracks on the borehole walls to a certain extent. When the spacing between blastholes detonated successively is ten times of the blasthole diameter, it is most conducive to forming straight and coalesced fractures;
2. Peak tensile stress on elements between blastholes detonated successively increases with the growing in-situ stress, which, however, does not change the change trend of the peak tensile stress;
3. The principal in-situ stress conditions exert significant influences on the propagation of blasting-induced cracks. To ensure blasting-induced cracks to propagate along the line connecting the blastholes, there is the optimal in-situ stress condition corresponding to the same blasting parameters. That is, when the in-situ stress reaches a certain value, the contour shaping effect is mainly affected by blasting parameters and geological conditions.

## Discussion

Although this study has resulted in some valuable conclusions, there are still some issues that warrant further investigation.

1. Due to the limitations of experimental conditions, most of the research on blast-induced crack propagation was conducted using numerical simulation methods. While numerical simulations can present more regular results, the research work ultimately needs to serve practical engineering the more fully to realize its value. In the future, the numerical simulation results can be validated and optimized through laboratory and on-site experiments, and relevant parameters can be improved to obtain widely applicable techniques;
2. The precise blasting excavation of deep underground caverns requires consideration of various complex factors. Other geological conditions, such as the distribution of joints and fractures, also play a crucial role in the shaping effect of blast-induced cracks. In the future, further consideration will be given to the influence of complex geological conditions on SCFB, with the aim of optimising blasting parameters and reducing the extent and severity of damage to the surrounding rock.

## Data Availability

The datasets generated during and analyzed during the current study are available from the corresponding author on reasonable request.
